# Protocol for a retrospective, controlled cohort study of the impact of a change in Nature journals’ editorial policy for life sciences research on the completeness of reporting study design and execution

**DOI:** 10.1007/s11192-016-1964-8

**Published:** 2016-05-11

**Authors:** Fala Cramond, Cadi Irvine, Jing Liao, David Howells, Emily Sena, Gillian Currie, Malcolm Macleod

**Affiliations:** Centre for Clinical Brain Sciences, University of Edinburgh, Edinburgh, Scotland, UK; School of Medicine, University of Tasmania, Hobart, TAS Australia

**Keywords:** Risk of bias, Reporting, Methodological quality, Study design, Reporting guidelines

## Abstract

In recent years there has been increasing concern about the rigor of laboratory research. Here we present the protocol for a study comparing the completeness of reporting of in vivo and in vitro research carried in Nature Publication Group journals before and after the introduction of a change in editorial policy (the introduction of a set of guidelines for reporting); and in similar research published in other journals in the same periods.

**Electronic supplementary material:**

The online version of this article (doi:10.1007/s11192-016-1964-8) contains supplementary material, which is available to authorized users.

## Background

Few publications describing in vivo research report taking measures which might reduce the risk of bias in their findings (Ioannidis et al. 2014; Macleod et al. 2015), and those which do not report such measures give inflated estimates of biological effects (Crossley et al. 2008; Hirst et al. 2014). Measures which might improve the quality of reports of in vivo research have been proposed (Kilkenny et al. 2010; Landis et al. 2012) and while these have been endorsed by a large number of journals there is evidence that this endorsement has not been matched by a substantial increase in the quality of published reports (Baker et al. 2014).

Poor replication of in vitro molecular and cellular biology studies has also been reported (Begley and Ellis 2012; Prinz et al. 2011) and linked in part to poor descriptions of the experimental and analytical details.

In May 2013 Nature Journals introduced a change in editorial policy which required authors of submissions in the life sciences to complete a checklist indicating whether or not they had taken certain measures which might reduce the risk of bias and to report key experimental and analytical details; and in their submission to detail where in the manuscript these issues were addressed (Anon 2013). The purpose of this study is to assess any impact of this change.

## Study design

1.1. *Aim* To determine whether the implementation of a checklist for submissions has been associated with improved reporting of measures which might reduce the risk of bias.
1.2. *Population* Published articles accepted for publication in Nature journals, which describe research in the life sciences and which were submitted after May 1st 2013 and before November 1st 2014.
1.3. *Intervention* Mandatory completion of a checklist at the point of manuscript revision.
1.4. *Comparator* Published articles accepted for publication in Nature journals in the months preceding May 2013, which describe research in the life sciences.
1.5. *Outcome* Change in proportion of published studies which report measures which might reduce the risk of bias.

## Identification of relevant manuscripts

### Nature publications

One individual was specifically employed by Nature to select manuscripts based on a pre-defined criteria for inclusion. Following this, a Nature editorial administration manager reviewed selected manuscripts against the inclusion criteria and found that some manuscripts (fewer than 10 %) had been incorrectly included; they replaced these with manuscripts that they selected according to the inclusion algorithm. Across all participating Nature publications (see “Appendix 1”) that describe primary research in the life sciences they identified papers accepted for publication with an initial submission date later than May 1st, 2013. Beginning with the current issue (volume corresponding to year 2015), they worked backwards in time, ensuring the submission date was after 1st May 2013, collecting papers until the number of studies required was reached (“Post intervention” group). They then used a similar process to identify papers submitted for publication before 1st May 2013, starting with the May 2013 issue and working backwards, ensuring that the date of submission was after 1st May 2011 (“pre-intervention” group). For each group, the intention was that 40 papers should be selected from Nature and 20 from each of the other titles. This would provide a total of 220 in each group and thus allow for any papers that did not fit the study to be excluded while ensuring the study includes 200 papers per group. The selection criteria, in addition to the dates of submission, were:

- the description of in vivo research (manuscripts that contain at least one non-human animal experiment, including rodents, flies, worms, zebrafish etc.) or in vitro research;
- journal of publication of Nature, Nature Neuro; Nature Immunology; Nature Cell Biology, Nature Chemical Biology, Nature Biotechnology, Nature Methods, Nature Medicine, or Nature Structural and Molecular Biology;
- for the post intervention group, similar proportions for country of address for correspondence and journal.

Where no pre-intervention period match could be found with a submission date after 1st May 2011 (i.e. in the 2 years leading to the change in policy) then the non-matched post intervention publication was excluded from analysis and a replacement post intervention publication selected, as above, with a matching pre-intervention publication then identified, as described above. Publications describing research involving only human subjects were not to be included.

The published files corresponding to the publication pdfs (including the extended methods section, extended data and other supplementary materials) will be used to generate pdfs for analysis; these study pdfs will not include author names or affiliations, date, volume or page number; or any references (to allow a blinded assessment of outcome). These study pdfs will be presented for analysis in random order with a unique study identifier. This will be done by the Nature editorial administration manager; Nature editors and publishers will have no role in selecting the study manuscripts. All studies to be included will be listed in an MS Excel file. Each will then be allocated a random number between 0 and 1 using the RAND() command. They will then be sorted according to this number and a unique identifier corresponding to their position in this new sequence will be allocated. These studies, with this unique identifier, will be batch uploaded to a study computer [the Edinburgh Microsoft Access reporting quality scoring system (MARQSS)]. Information on group membership will be retained by the Nature editorial administration manager. Only once data analysis is complete will the coded group allocation (A and B) be revealed to the study team, along with coded research area, research area and country of origin. The study team will then prepare two reports, one to be used if group A is pre-change, and one to be used if group B is pre-change.

### Sister publications

To investigate whether the changes apply only to Nature publications or whether there has been an increase in the general scientific literature of the proportion of published studies which report measures which might reduce the risk of bias, we will match all the Nature publications included with a sister publication (also selected from before and after May 2013 as described above) with the following methodology:

1. Using PubMed, enter the Nature publication title.
2. Add the “related citations for PubMed” result to the search builder.
3. In the second line search field “Date of publication” for related articles in the same calendar month (M0) and year (YYYY/MM).
4. Search.
5. In the results, start with the first result returned and establish that it was not published in an NPG Journal (“Appendix 1”).
6. That being the case, then apply the study inclusion criteria (2.2.1), ensuring that there is a match on the in vivo/in vitro status between Nature publications and non-Nature publications.
7. If the manuscript fulfils 5 and 6 select it for the study and retrieve the pdf. If not available from institutional subscriptions, seek the pdf of the paper and any supporting materials through an interlibrary loan or from the authors.
8. Save a pdf file comprising the main manuscript and supporting information with a name in the form NPG <xxx>_pair_nonredact.
9. If the manuscript fails 5 or 6 repeat the search with the date of publication extended to 1 month earlier and 1 month later (M − 1 to M + 1).
10. Repeat steps 5 through 9 until a matching publication is found.
11. Record the difference in calendar months relative to the date of publication of the index NPG article.

### Inclusion and exclusion criteria

Manuscripts describing research in the life sciences, categorised as

1. those describing in vivo research (manuscripts that contain at least one non-human animal experiment, including rodents, flies, worms, zebrafish etc., and where either the exposure of interest, or the outcome, or both, are determined in whole living animals);
2. those describing exclusively vitro research (exclusively molecular and cellular biology);
3. those presenting both in vivo and in vitro experiments, as described above.

Publications describing research involving only human subjects alone will not be included—if there are animal studies included, the publication can still be used.

### Redaction

To blind reviewers a redaction process will be carried out by a scientist in another institution:

1. Load the pdf to Adobe acrobat professional.
2. Identify date identifying information including publication date; volume and issue number; grant and funding information; and all references, including those which are in-line [i.e. (Smith et al. 2015)], those which are more integrated (i.e. “In a 2015 study by Smith and co-workers), and also in-line acknowledgements (i.e. “Reagent x kindly donated by R. Smith”). Additionally, any reference to years in the text was removed (i.e. “During the 1980s…”, “2015 census data…”).
3. Use the redaction tool to redact these data.
4. Save the file with a name in the form NPG<xxx>_pair_redact.
5. Upload this redacted file.

### Outcome assessment

Manuscripts will be scored by two independent reviewers blinded both to intervention status (before or after the change in editorial policy) and to the scores from the other reviewer. While the source of the publication (NPG or not, which NPG journal) will not be redacted, this will likely be apparent to most reviewers anyway from the typeface, layout, house style etc. Discrepancies will be resolved by a third reviewer who will be blinded to the identity, and the scores of the previous reviewers.

We will recruit individuals experienced in the critical appraisal of published materials (through for instance involvement with previous systematic reviews). Reviewers will receive training using an online platform, supported by a training manual (supplementary material). They will be presented with manuscripts to score, and their assessment compared with a “gold standard” derived following review of a set of manuscripts each scored in house by the CAMARADES team. Once their concordance with that gold standard is greater than 80 % for three successive manuscripts they will be considered to have been trained to a sufficient standard.

To score a manuscript they will log on to MARQSS, and will be allocated the next manuscript requiring to be scored for the first time. If all manuscripts held in MAQRSS at that time have already been scored once, then the reviewer will be allocated a manuscript for second screening. MAQRSS will ensure that reviewers do not receive for second review a manuscript for which they performed the first review. Once manuscripts have been scored twice MARQSS will compare those scores, item by item, and flag those manuscripts where there is a discrepancy. These discrepancies will then be resolved by a third, senior reviewer.

Where a manuscript describes both in vivo and in vitro research, data will be extracted for both. Where there is more than one experimental design under each of these headings, quality criteria must be reported for all experiments to be awarded the point, however some checklist items will have a ‘partially’ option.

Monitoring of outcome assessment after 10 % of manuscripts have been scored and adjudicated; we will review performance and if there are questions that are highly represented in those resulting in disagreements we will review the training materials and amend them as appropriate.

### Primary outcome measure

The proportion of publications in the intervention group describing in vivo research that meet the Landis criteria (item numbers #2, #3, #4, #5 of “Appendix 2”). For the purpose of this study, meeting the criteria means that for a study in which the parameter is relevant, it was reported as being performed or as not being performed. The evaluation principle is to determine if someone with reasonable domain-knowledge can understand the parameters of experimental design sufficiently to inform interpretation. These metrics will not be applied to exploratory studies, defined for this purpose as a study where hypothesis testing statistical analyses were not reported.

### Secondary outcome measures

#### In vivo research

1. The change in prevalence of reporting of all of the Landis criteria (#2,3,4 and 5 together).
2. For 3 other items identified in Table 1 and for the 4 individual components of the Landis checklist (#1,2,3,4,5,7,9), the proportion of publications in the intervention and comparison groups considered to meet all of the relevant Nature checklist criteria.

**Table 1:** Detecting an editorially significant change or prevalence for specified items of the checklist

Type of study^a^		Editorially significant change or prevalence	
Category of requirement	Operationalized checklist items	In vitro studies	Animal studies
Transparency in figures and statistical description	#1	80 % compliance	80 % compliance
Data deposition	#9	80 % compliance	80 % compliance
Animals description	#7	N/A	80 % compliance
“Landis 4”	#2, 3, 4, 5	15 % increment	80 % compliance
Reagents description (Ab and Cell line)	#6	15 % increment	15 % increment
Code availability	#10	15 % increment	15 % increment

^a^The unit of assessment will be considered to be the publication; while editorial intervention could occur at any level, any impact of this would be mediated through an effect on the author, and this is therefore the smallest unit to which an intervention might be allocated at random. Where there is more than one experimental design under each of these headings, quality criteria must be reported for all experiments to be awarded the point, however some checklist items will have a ‘partially’ option. However, if there are two in vivo or in vitro experiments, one randomized non blinded one blinded non randomized, for the primary outcome we will consider the paper to be both randomized and blinded
3. For 2 other items identified in Table 1 (#6, 10), the change in the proportion of publications meeting that criteria between the comparison group and the intervention group.

#### In vitro research

1. The proportion of publications in the intervention group describing in vitro research that meet the Landis criteria (#2, #3, #4, #5 in the “Appendix 2”).
2. For 3 other items identified in Table 1 and for the 4 individual components of the Landis checklist (#1,2,3,4,5,9), the proportion of publications in the intervention and comparison groups considered to meet all of the relevant Nature checklist criteria.
3. For 2 other items identified in Table 1, the change in the proportion of publications meeting that criteria between the comparison group and the intervention group.

The papers that contain both in vitro and in vivo research will be scored for each type of research independently and will contribute to both of these secondary outcomes. For each secondary outcome the denominator for calculation of proportions will total number of papers reporting at least one in vivo or at least one in vitro experiment, respectively. We will also evaluate whether papers reporting both in vivo and in vitro results are more or less likely to be compliant on each secondary outcome measure.

### Tertiary outcome measures

1. The proportion of publications in the intervention and comparison groups considered to meet all of the relevant Nature checklist criteria.
2. For each of the individual components of the Nature checklist, change in the proportion of publications meeting that criteria between the comparison group and the intervention group.

## Statistical considerations

### Power calculations

The operationalized Nature checklist comprises 77 items arranged under 19 headings. These items are very different in their form and, probably, in their relative importance. Not all will be relevant to all studies. Further, complete compliance with the Nature guidelines, while the desired outcome, is likely to remain uncommon; it would also be interesting to observe differences in the reporting of 27 key components (items under 1,2,3,4,5 and 7) of the checklist for animal studies, as well as relatively novel requirements (such as items under 6 and 10). The power calculations which follow are for in vivo research; for in vitro research they will be broadly similar.

### Primary outcome (see above)

The Nature editorial team indicate that for the primary outcome they wish to know whether compliance with the “Landis 4”, in aggregate, reaches 80 %. Table 2 shows the upper limits of observed compliance which might be found to be significantly lower than 80 %, at different levels of power, for different numbers of observations, using a one-sample proportion Wald test with *p* < 0.05. 100 manuscripts would deliver 70 % power, 150 manuscripts would deliver 80 % power and 200 manuscripts would deliver 90 % power to detect compliance 10 % lower than this.

**Table 2:** Upper limits of observed compliance at different power cut-offs

Sample size (per group)	0.70 power (%)	0.80 power (%)	0.90 power (%)
100	70	68	66
150	72	71	69
*200*	*73*	*72*	*70*
250	74	73	72

### Secondary outcome (in vivo research)

Table 3 shows the changes in aggregate reporting of the Landis 4 criteria identified at different levels of power using a one sided two sample Chi squared test (STATA) (4 comparisons, *p* < 0.01). Italic values are where an increase of 15 % or less is detected.

**Table 3:** Changes in aggregate reporting of the Landis 4 criteria identified at different levels of power

Prevalence before (%)	Prevalence detected (0.70 power) (%)	Prevalence detected (0.80 power) (%)	Prevalence detected (0.90 power) (%)
10	*20*	*21*	*23*
20	*32*	*34*	*36*
30	*44*	*45*	47
40	*54*	56	58
50	*64*	66	67

### Secondary outcome (in vitro research)

Table 4 shows the upper limit of observed compliance with the Landis 4 criteria which would be identified as being significantly less than 80 % detected with different power using a one-sample proportion Wald test: (9 comparisons, *p* < 0.0051).

**Table 4:** Upper limits of observed compliance at different power cut-offs

Sample size (per group)	0.70 power (%)	0.80 power (%)	0.90 power (%)
100	65	64	61
150	68	67	65
*200*	*70*	*69*	*67*
250	71	70	69

### Secondary outcome (see 3.2 (III))

Table 5 shows the power to detect an absolute 15 % increase in the reporting of each of #6 and #10, depending on the prevalence in the control group, using a one sided two sample Chi squared test (STATA), with 200 manuscripts in each group (9 comparisons (with 3.2.1.2, *p* < 0.0051). Italic values are where the power is >0.80.

**Table 5:** Power required to detect an absolute 15 % increase in the reporting of items #6 and #10 of the Landis criteria items

Prevalence before (%)	Prevalence after (%)	Power
5	20	*0.98*
10	25	*0.92*
15	30	*0.85*
20	35	0.79
25	40	0.74
30	45	0.70
35	50	0.68
40	55	0.67
45	60	0.67
50	65	0.68

### Statistical analysis plan

We will use the Wald test to calculate the proportion (and 95 % confidence intervals) of studies meeting the primary outcome, and for other outcomes where the proportion of manuscripts meeting individual components of the checklist is reported (3.2.1.2). For secondary outcomes, the 95 % confidence boundaries will be adjusted to take account of the number of comparisons drawn (9 comparisons, *p* < 0.0051, so 99.49 % CIs). We will use Pearson Chi squared tests to test the significance of differences between groups for other secondary and tertiary outcomes, again with Holm-Boneferroni adjustment of critical *p* values to account for multiple testing. We will also conduct an interrupted time series analysis using itsa in Stata, for the change in the proportion of publications meeting the primary outcome measure with dates of submission before or after May 2013 in the “treated” (NPG) group compared with the “control” (matched) group.

We will conduct sub-group analyses in groups defined by country of origin; categorisation of research; and whether the study is predominantly in silico; in vitro; in vivo; or involves human subjects.

## Ethical approval

This study is a retrospective quality audit rather than a randomised trial. As such there are no ethical concerns.

## Role of Nature in data analysis and data ownership

The study dataset will belong to the investigators, and all decisions relating to data analysis and publication will be taken by the steering committee and will be independent of Nature. Once the manuscript is written, it will be shared with NPG and they will be invited to correct any errors of fact.

## Publication policy

The entire set of derived data will be made available in a publically accessible, curated database within 12 months of completion of the study, or upon publication of the results of the study, whichever comes first. The study protocol will be lodged in a curated database. Because of the potential for conflict of interest the manuscript describing this work will not be submitted to any of the Nature journals but to a different journal.

Authorship of the main study report will include all those who have participated in the study design, planning, data collection or analysis.

## Committees

The committee structure is defined to allow separation of interests.

### The study steering committee

The study steering committee is responsible for strategic decisions regarding the study; and for data analysis, and for writing the first draft of the study report. It comprises Malcolm Macleod (University of Edinburgh, Chief Investigator and Chair), Emily Sena (University of Edinburgh), David Howells (School of Medicine, University of Tasmania).

### The study management committee

The study management committee is responsible for ensuring the smooth running of the study, and at the outset comprised the study steering committee along with Veronique Kiermer (Nature). Dr Kiermer resigned from the study management committee in mid-2015 when she left NPG, and was replaced by Dr. Sowmya Swaminathan.

### Electronic supplementary material

Below is the link to the electronic supplementary material.

Supplementary material 1 (PDF 599 kb)

## Appendix 1: List of Nature named journals

- Nature
- Nature Biotechnology
- Nature Cell Biology
- Nature Chemical Biology
- Nature Communications
- Nature Genetics
- Nature Immunology
- Nature Medicine
- Nature Methods
- Nature Neuroscience
- Nature Structural and Molecular Biology
