# Supplementary material for: Protocol for a retrospective, controlled cohort study of the impact of a change in Nature journals’ editorial policy for life sciences research on the completeness of reporting study design and execution
Source: Scientometrics. 2016 May 11;108:315–28. doi: 10.1007/s11192-016-1964-8 (PMC4909786; doi:10.1007/s11192-016-1964-8)
Supplement: Supplementary file 1 — Supplementary material 1 (PDF 599 kb) [file 11192_2016_1964_MOESM1_ESM.pdf]

Nature **P**ublication **Q**uality **I**mprovement **P**roject TRAINING RESOURCE

This is not an exhaustive guidance but can be used for reference for some of the common issues that may be faced throughout the review process.

Importantly, where the reviewer feels that the item is only partially described in the manuscript, and there is no **'Partially'** option then they should select **'No'.**

0.1 Experiment type

Does this publication describe in vivo experiments, in vitro / ex vivo experiments or both?

If the authors report their experiments as in vivo or ex vivo/in vitro then use their categorisation when reviewing. Otherwise, select the study type you believe the paper to be describing:

- **In vitro:**
  Latin: in vitro = "within the glass"
  Studies that are in vitro are performed on cells or biological molecules studied outside their normal biological context; for example proteins are examined in solution, or cells in artificial culture medium.
    - **Ex vivo:**
        - Ex vivo refers to experimentation done in or on tissue from an organism in an external environment with the minimum alteration of natural conditions. This should be classified as in-vitro.
    - **Human:**
        - Note that this can also include in vitro or ex vivo study of human samples (also see 0.2).
- **In vivo:**
  Latin: in vivo = "within the living"
  Studies that are in vivo are those in which the effects of various biological entities are tested on whole, living organisms. In the situation that samples are taken from the animals after the experimental interventions have been carried out in vivo (i.e. blood samples, brain slices, DNA from transgenic animals) this would still be classified as an in vivo only study. Similarly, if the experiment involves the creation of transgenic animals and no further interventions are carried out ex vivo/in vitro then this would also be in vivo.

If a study describes both in vivo and ex vivo experiments select **'Both'**. In the case that you believe that the study does not describe in vitro/ex vivo or in vivo study please select **'Neither'** and leave a comment to justify this response.

0.2 Human related

Does this publication include human study?

Select **'Yes'** if the study includes human study (i.e. human subjects or tissue of human origin) which may include human samples such as tissue slice (ex vivo or in vitro). It would not be classified as a human study simply if the study used cell lines of human origin and **'No'** should be selected in this case.

0.3 Computer code

Does this publication include computer code?

If computer code was used to generate results that are central to the paper's conclusions, authors should include a statement in the Methods section under for example ***"Code availability"*** to indicate whether and how the code can be accessed and any restrictions. This could include for

instance a paper that uses (mathematical) modelling techniques. If it is clear that code has been used for the publication select **'Yes'** and if not select **'No'.**

This <u>does not</u> refer to *in silico* experiments.

0.4 Comments

Do you have other comments? If yes, please leave them here.

If you have any comments regarding the interpretation of the paper please leave these here. You may need to go back to this page once you have finished scoring the manuscript to note any issues you may have had or to flag any points you wish to make.

1. Figures and statistical representation of data

The following **1.1 – 1.4.6** items should be found either in the **figure legends or the methods section (please also search through supplementary materials)** of the manuscript.

In the case where you have selected **'Both'** i.e. the manuscript includes in vitro and in vivo experiments be sure to check that the following items apply to each one as there may be instances where the in vivo experiments reported correctly and therefore would get a **'Yes'** for an item whereas the in vitro failed to report it thus getting a **'No'.**

1.1 Sample size numbers

The legend or the main text report the exact sample size (n) for each experimental group/condition, given as a number, not a range?

To answer **'Yes'** for this item there needs to be **clear** sample size number reported for each group and this should be also be indicated in the relevant sections of the manuscript (in the methods, figure legends, and/or in supplementary materials) when appropriate. On the other hand if there is any ambiguity, you would select **'No.'** Select **'No'** when they have only reported the size of the treatment group and not the control group or if n values are only reported for one experiment when others experiments have been carried out with different groups.

For in vitro studies for example when reporting gene expression, western blot results etc. the authors should report n values for either the corresponding control and treatment group of animals or the sections/samples used (see also ***section 1.2*** on technical and biological replicates).

1.2 Technical or biological replicates

A description of the sample collection allowing the reader to understand whether the samples represent technical or biological replicates (including how many animals, litters, cultures, etc.)?

Replication implies having at least one experimental unit per treatment condition, and is necessary in order to permit estimation of experimental error or variance. Biological replication refers to the number of independent biological (experimental) units assigned to each treatment condition whereas technical replicates arise from repeated sampling of the same experimental unit and will therefore be correlated (Cui and Churchill, 2003).

Biological replicates are when the same type of organism is grown/treated under the same conditions. For example, if one was performing a cell-based study, then different flasks containing the same type of cell (and preferably the exact same lineage and passage number), which have been grown under the same conditions, could be considered biological replicates of one another.

Technical replicates would be when the exact same sample (after all preparatory techniques) is analysed multiple times. The point of such a technical replicate would be to establish the variability (experimental error) of the analysis technique (mass spectrometry, LC, etc.), thus allowing one to set confidence limits for what is significant data. This is in contrast to the reasoning behind a biological replicate, which is to establish the biological variability, which exists between organisms, which should be identical. Knowing the inherent variability between "identical" organisms allows one to decide whether observed differences between groups of organisms exposed to different treatments is simply random or represents a "true" biological difference induced by such treatment.

Table 1 | Replicate hierarchy in a hypothetical mouse single-cell gene expression RNA sequencing experiment

	Replicate type	Replicate category[a]
Animal study subjects	Colonies	B
	Strains	B
	Cohoused groups	B
	Gender	B
	Individuals	B
Sample preparation	Organs from sacrificed animals	B
	Methods for dissociating cells from tissue	T
	Dissociation runs from given tissue sample	T
	Individual cells	B
	RNA-seq library construction	T
Sequencing	Runs from the library of a given cell	T
	Reads from different transcript molecules	V[b]
	Reads with unique molecular identifier (UMI) from a given transcript molecule	T

[a]Replicates are categorized as biological (B), technical (T) or of variable type (V). [b]Sequence reads serve diverse purposes depending on the application and how reads are used in analysis.

Therefore, if it is clear that the experiments include replicates and it is clear that they are either biological of technical select **'Yes'** (note: the authors may not have explicitly referred to biological or technical replicates but if enough detail given then the paper can still be awarded this).

Select **'No'** if the manuscript describes replicates but it is not clear whether they are biological or technical replicates.

If the experiment does not describe either biological or technical replicates select **'Not Applicable'.**

For more in detail information on the table above and replicates please see the following publication: http://www.nature.com/nmeth/journal/v11/n9/pdf/nmeth.3091.pdf

1.3 Replication of experiments

A statement of how many times the experiment shown was replicated in the laboratory?

If all of the experiments described are stated to have been replicated, and the number of times it was replicated is reported, select **'Yes'.** When replication is reported but not explicitly for all relevant experiments or the number of times that it was replicated is not stated then select **'Partially'** and if none of the experiments were replicated select **'No'.**

Often the replication of experiments will be described as follows:

- …experiments were carried out in triplicate or duplicate etc. or,
- …the experiment was repeated twice etc.

Select **'Not Applicable'** if an experiment was not repeated, but they performed a sample size calculation and the experiment was adequately powered.

1.4 Definitions of statistical methods and measures

1.4.1 Description of non-common tests

If not a very common test, is the test described in the methods section? (Note that common tests are considered as t-test, simple χ2 tests, Wilcoxon and Mann-Whitney tests, and any form of ANOVA testing)

When a study uses a more complex statistical test, stated in either the methods or results that is not one of the tests listed above there should be some explanation of its application described in the methods section. Any type of

You would select **'Yes'** for example if it is described how the test was used and for which groups:

- …followed by **Newman-Keuls post hoc tests** to determine differences among groups for interactions between n-3 PUFAs or sevoflurane and cleaved caspase-3 activation, BrdU quantification, or neurobehavioral tests.

- Two-tailed t-test and two-way ANOVA with post-hoc **(Tukey)** test [SAS software (Cary, NC, USA)] were used to compare the differences between groups.

If there have been various tests used and only a proportion have been described sufficiently then select **'Partially'**. If only common tests have been reported in the manuscript select **'Not Applicable.'** Select **'No'** when non common tests have not been described i.e. their usage is not clear.

1.4.2 Tests reported as one-sided or two-sided?

If the statistical test used is a t or z test, was this reported as one sided, two sided?

Studies should have reported whether their statistical test is one sided or two sided (or one tailed or two tailed) if they use the following tests:

- t test (also known as Student's t-test),
- z test

If this is the case you should select **'Yes'** in the drop down options. If they have however used these tests and not stated the tests as one sided or two sided select **'No.'** If tests other than a t-test or z-test have been used then select **'Not applicable'.**

1.4.3 Multiple comparisons

Are there adjustments for multiple comparisons where this is appropriate?

When more than one statistical comparison is performed (e.g. drug effects at multiple times points), some p values may be significant by chance alone. A multiple comparison test takes into account the number of comparisons when calculating the p value. Possible statistical tests for multiple comparisons include:

ANOVA

Analysis of variance (ANOVA) is a collection of statistical models used to analyse the differences among group means and their associated procedures (such as "variation" among and between groups). The ANOVA test tells you whether you have an overall difference between your groups, but it does not tell you which specific groups differed - post hoc tests do and may include one of the ones mentioned below.

If the authors state that this test shows a difference between identified groups then this is not sufficient information and a post hoc test needs to be stated. If no post hoc test is stated in this case then select **'No'.**

Post hoc tests for multiple comparisons

If the decision on what comparisons to make is withheld until after the data are examined, the following procedures can be used:

- Bonferonni correction
- Tukey's method
- Scheffé's Method

Other multiple comparison tests

- Dunnet's test
- Tamhane correction
- Sidak correction
- Newman-Keuls Multiple Comparison Test
- Steel's test

If the manuscript describes multiple comparisons and reports an appropriate test select **'Yes'** if multiple comparisons are carried out but no test is described select **'No'.** If there are no multiple comparisons carried out then select **'Not Applicable'.** Testing for multiple comparisons is appropriate when an investigation/experiment compares several different factors between two groups. Consider the efficacy of a drug in terms of the reduction of any one of a number of disease symptoms: as more symptoms are considered, it becomes more likely that the drug will appear to be an

improvement over existing drugs in terms of at least one symptom, therefore increasing the risk of getting a false positive result. Tests of multiple comparisons take this into account.

1.4.4 Statistical test results

Are the statistical test results (e.g., P values, F statistic) presented?

For p values, this includes whether they report exact numbers or cut offs i.e. for p values <0.05 or <0.01. There should be the reporting of t-values for t-tests, R values for correlation & regression, Chi-square (χ^2) value for chi-squared test etc. The test statistics for each test should be reported, if they are reported for each statistical test used, select **'Yes'** if not select **'No'.** If these tests have not been used then select **'Not Applicable'.**

Examples of appropriate test result reporting:

- **T-test:** There was a significant difference in the scores for sugar (M=4.2, SD=1.3) and no sugar (M=2.2, SD=0.84) conditions; t (8)=2.89, p = 0.020.
- **ANOVA:** There was a significant effect of amount of sugar on behaviour remembered at the p<.05 level for the three conditions [F(2, 12) = 4.94, p = 0.027].
- **Regression:** Risk factor 1 significantly predicted depression scores, b = -.34, t(225) = 6.53, p < .001. Risk factor 1 also explained a significant proportion of variance in depression scores, R^2 = .12, F(1, 225) = 42.64, p < .001.
- **Correlations:** you simply report the r value and, if appropriate, the corresponding p-value. e.g., "X and number of Y were correlated with r = -0.70, p = .02"

If only p-values are reported along with only a mention of which statistical test was used and they do not also report the appropriate test results e.g. t values, R values etc., select **'Partially'.**

It is not possible to list all possible tests here so for any other statistical tests please check if other test results are required other than a p value for example only a p value required for an exact Fisher's test (i.e. **'Yes'** if p values then presented). It would be helpful if you leave a comment to justify your selection.

1.4.5 Summary estimates

Are the summary estimates defined as a median or average?

Summary estimates may be defined as median, averages, and/or means. Select **'Yes'** if they report which summary estimate they used and report it appropriately in the methods and/or in the legends, select **'No'** if they do not. Authors should however report the summary estimates where appropriate, if in the methods they state that '*Data are shown as mean+/- SEM or S.D*' one can assume this is true for all results and you should select **'Yes'** however, is this not explicit then the mean should be reported specifically where appropriate in results i.e. in figure legends etc. This may be **'Not Applicable'** for instance if not statistical analyses were carried out or the n value is 1 etc.

1.4.6 Error bars

Are the error bars defined as standard deviation (s.d.), standard error of the mean (s.e.m.), or confidence intervals (c.i.)?

Select **'Yes'** if they define their error bars in the methods and/or the legends where appropriate. In the case where they have stated in the methods that all values are reported and graphed as mean +/- s.e.m. then if this is missing from the legends it can be assumed that error bars represent s.e.m. and **'Yes'** should be selected. If this is not explicit and if some or all error bars are not defined then select **'No'.** If the authors have not reported their results graphically with error bars then select **'Not Applicable'.**

1.5 Implementation of statistical methods and measures

The following information can be mentioned in **methods, results or legends** (including any **supplementary materials**).

1.5.1 Meeting assumptions of tests

Do the authors show that their data meet the assumptions of the tests (e.g., normal distribution)?

Since a number of the most common statistical tests rely on the normality of a sample or population, it is often useful to test whether the underlying distribution is normal, or at least symmetric. This can be done via the following approaches:

- Review the distribution graphically (via histograms, boxplots, QQ plots)
- Analyse the skewness and kurtosis
- Employ statistical tests (esp. Chi-square, Kolmogorov-Smironov, Shapiro-Wilk)

Typical assumptions are:

- **Normality: Data have a normal distribution**
- Homogeneity of variances: Data from multiple groups have the same variance (i.e. Levene's test)
- Linearity: Data have a linear relationship.
- Independence: Data are independent.

If the authors state that their data meet the assumptions of their tests select **'Yes'** and select **'No'** if this is not stated.

If not statistical analyses are carried out then this would be **'Not Applicable'.**

1.5.2 Variation within each group of data

Is there an estimate of variation within each group of data?

In simplistic terms, variance measures how far a set of numbers are spread out. A variance of zero indicates that all the values are identical. Possible statistical tests for variance within groups include:

- Standard deviation (s.d.) – either presented in raw values in text or figure legend, or as s.d. error bars on a graph
- s.e.m error bars presented and the exact n values of groups
- Range or Interquartile Range
- Variance – a measure of the sigma squared value

If there is a measure of variation (e.g. s.d.) for each group – e.g. control, intervention 1 and intervention 2, then select **'Yes'**. If the estimation of variance i.e. s.d. of only one group is reported, or i.e. s.d. is not reported for any groups, select **'No'.** Select **'Not Applicable'** if for example there is an n of 1, for observational assessments etc.

Note *that s.e.m is not a measure of variation within the group, the standard error of the sample is an estimate of how far the sample mean is likely to be from the population mean, whereas the standard deviation of the sample is the degree to which individuals within the sample differ from the sample mean. However if s.e.m is reported with sample sizes, the variation can be imputed and so select* ***'Yes'***.

1.5.3 Variance between groups being compared

Is the variance similar (difference less than two-fold) between the groups that are being statistically compared?

This can be ascertained in the same way as establishing within group variance (e.g. looking at s.d. or variance statistic), however this requires an additional step of comparison. This may require a judgement of s.d. error bars on graphs presented if raw data are not available. Infrequently, authors might state that e.g. a Levene's test, Barlett's test or F-test of equality of variances has been used to

assess whether there is equality of variances for the two (or more) groups. In this situation, select **'Yes'**.

If authors state that, for example, Levene's or Barlett's test was not significant then the variability in the two (or more) groups/conditions are roughly similar, a difference of less than two-fold i.e. select **'Yes.'** However, if Levene's or Bartlett's test is significant, indicating that there is variability, the difference may be less than 2 fold therefore select **'Not able to judge.'** If it requires a judgement of s.d. error bars on graphs try to measure as best as you can but if it is too difficult or they have used s.e.m with n values select **'Not able to judge'.** Select **'Not Applicable'** if 1.5.2 is 'Not Applicable' or for example there is an n of 1, for observational assessments etc.

Select **'No'** if the variance is clearly more than two fold either graphically or in the text/legend.

2. Sample size

2. Appropriately powered study

Does the manuscript describe how the sample size was chosen to ensure adequate power to detect a pre-specified effect size?

Sample size determination is a process of choosing the number of observations or replicates to include in a statistical sample. The sample size is an important feature of any empirical study in which the goal is to make inferences about a population from a sample. In practice, the sample size used in a study is determined based on the expense of data collection, and the need to have sufficient statistical power.

The power or sensitivity of a binary hypothesis test is the probability that the test correctly rejects the null hypothesis (H0) when the alternative hypothesis (H1) is true. It can be equivalently thought of as the probability of accepting the alternative hypothesis (H1) when it is true – that is, the ability of a test to detect an effect, if the effect actually exists.

To select **'Yes'**, studies should give an explanation of their sample size – this should be a statistically based calculation to justify their sample size number. If the authors mention that their study was appropriately powered but do not present how this was quantified select **'No'.** Select **'Partially'** if only for example 1 out of two experiments were powered to detect an effect (i.e. this could be a sample size calculation for only one out of multiple independent in vivo experiments or in vitro experiments).

It is unlikely this would be **'Not Applicable'** in the case that you believe this to be the case please explain in the comments section.

Please paste the relevant and corresponding text in the comments section if the answer to this item is either **'Yes'** or **'Partially'**.

2.1. Statement about sample size estimate

Does the manuscript include a statement about sample size estimate even if no statistical methods were used?

If the answer to 2 is **'Yes'** then select **'Not Applicable'** for this question.

If the answer to the above is **'No'** then the next step would be to see whether sample size estimates is mentioned at all and if there is a statement then select **'Yes'** if not select **'No'** as well.

Please paste the relevant and corresponding text in the comments section if the answer to this item is either **'Yes'** or **'Partially'**.

3. Exclusions

3. Exclusion of samples or animals from the analysis

Does the manuscript describe if samples or animals were excluded from the analysis?

If the authors provide any description statement about whether a sample or animal being excluded from an analysis select **'Yes.'** Also select **'Yes'** when the statement says that there were no data points excluded from the analysis**.** If no exclusions are described then select **'No'.**

Please paste the relevant and corresponding text in the comments section if the answer to this item is either **'Yes'** or **'Partially'**.

3.1 Defining exclusion criteria

Are exclusion criteria defined?

If there is an explanation of **why or in which situation** they would exclude animals or samples, select **'Yes'.** If they simply state an animal was excluded (without a reason) or if the n values change from the methods to the results with no explanation or if there is no mention of **why or in which situation** they would exclude animals or samples then select **'No'.** It is unlikely this would be **'Not Applicable'** in the case that you believe this to be the case please explain in the comments section.

Please paste the relevant and corresponding text in the comments section if the answer to this item is either **'Yes'** or **'Partially'**.

3.1.1 Pre-establishing exclusion criteria

Is it clear that the criteria were pre-established?

If the answer to 3.1 is **'No'** then select **'Not Applicable'** for this question.

This needs to be **explicitly stated** i.e. "pre-established criteria for exclusion include…" in the methods, it cannot be assumed that if it is described in the methods that it was pre-established. They may state that criteria were pre-defined and may reference a protocol and in this case select **'Yes'.** If you have selected **'Yes'** for 3.1 but it is not clear that exclusions were pre-defined then select **'No'.**

Please paste the relevant and corresponding text in the comments section if the answer to this item is either **'Yes'** or **'Partially'**.

4. Randomization

4. Method of randomization

Does the manuscript describe which method of randomization was used to determine how samples/animals were allocated to experimental groups?

Select **'Yes'** if a **method** of randomization has been described and used to allocate the samples or animals to their experimental group, for each experiment. **'Partially'** should be selected if randomization has only been done for a proportion of experiments reported. The absence of statement is taken to mean that no randomization has been carried out, select **'No'**. Select **'Not Applicable'** for instance if the randomisation was carried out in vivo and then exact same # of samples were used from these in vivo animals for ex vivo/in vitro experiments (e.g. # in same treatment / control groupings). It is unlikely this would be **'Not Applicable'** in the case that you believe this to be the case please explain in the comments section.

Please paste the relevant and corresponding text in the comments section if the answer to this item is either **'Yes'** or **'Partially'**.

4.1 Statement about randomization

Does the manuscript include a statement about randomization even if no randomization was used?

If the answer to 4.1 is **'Yes'** or **'Partially'** then select **'Not Applicable'** for this question.

Select **'Yes'** if a statement describing randomization even if simply to state that randomization was not possible was stated (this includes if randomization was stated but no methods were provided). In addition, if there is a statement about randomization but the method has not been described select **'Yes'.** Select **'No'** if 4 is **'No'** and there were no statements regarding randomization in the manuscript.

Please paste the relevant and corresponding text in the comments section if the answer to this item is either **'Yes'** or **'Partially'**.

5. Blinding

5. Method of blinding

Does the manuscript describe whether the investigator(s) was/were blinded to the group allocation during the experiment and/or when assessing the outcome?

Select **'Yes'** if blinding has been reported for either or both group allocation and/or when assessing the outcome (this should be done for each experiment / outcome). Select **'No'** if blinding is not described for any of these items and select **'Partially'** if they have only blinded for a portion of the experiments (either by blinding allocation or assessment of the outcome).

It is unlikely this would be **'Not Applicable'** in the case that you believe this to be the case please explain in the comments section.

Please paste the relevant and corresponding text in the comments section if the answer to this item is either **'Yes'** or **'Partially'**.

5.1. Statement about blinding

Does the manuscript include a statement about blinding even if no blinding was done?

If the answer to 5 is **'Yes'** or **'Partially'** then select **'Not Applicable'** for this question.

Select **'Yes'** if a statement describing blinding even if simply to state that blinding was not possible was stated. Select **'No'** if 5 is **'No'** and there were no statements regarding blinding in the manuscript.

Please paste the relevant and corresponding text in the comments section if the answer to this item is either **'Yes'** or **'Partially'**.

6. Reagents (in vivo)

6.1.1 Antibodies profiled for use in the system under study by citation

Has every antibody used in this manuscript been profiled for use in the system under study by either citation, catalog number, clone number or validation profile?

When only a proportion of all antibodies have been reported in an appropriate manner then select **'Partially'.**

Citation

Check that the citation details the antibody specified and its use in the appropriate assay/species (i.e. species used in manuscript matches species that it has been profiled for use in/reacts with) if this is the case select **'Yes'.** Similarly if the antibody name and supplier are reported and this can be

easily searched online (provided that the species used in manuscript matches species that it has been profiled for use in/reacts with) also select **'Yes'.**

If for example it has been profiled in a different species or the citation does not provide adequate information select **'No'.** If techniques are used such as immunohistochemical staining but the antibodies are not described select **'No'.** In the case that the references have been blacked out and you are unable to assess this please select **'Not able to judge'.** If the manuscript does not describe antibodies select **'Not Applicable'.**

Catalog number and/or clone number

Check that the catalog number or clone number details the antibody specified and its use in the appropriate assay/species (i.e. species used in manuscript matches species that it has been profiled for use in/reacts with) if this is the case select **'Yes.'** If for example it has been profiled in a different species or the catalog number or clone number does not provide adequate information select **'No'.** If the manuscript does not describe antibodies select **'Not Applicable'.**

Example of what catalog numbers may look like:

- For the detection of OCT4 we used two antibodies with similar results, BD **611203**, dilution 1:200, and Santa Cruz **sc-5279**, dilution 1:400; for NANOG, Novus **NB100 58842**, dilution 1:50; and for CDX2, Epitomics #2475-1, dilution 1:400.
  - Take Santa Cruz **sc-5279** as an example if you go online you will find a description as follows:

    Santa Cruz Biotechnology, Inc.'s Oct-3/4 (C-10) Antibody is a Mouse monoclonal antibody (clone C-10). The Oct-3/4 (C-10) Antibody was generated using POU Class 5 Homeobox 1, and pou5f1 as the antigen and it **reacts with Human, Rat, and Mouse**. This antibody has been shown to work in applications such as: Western Blot, Immunohistochemistry, ELISA, Immunofluorescence, Immunohistochemistry - fixed, Flow

Validation profile (e.g., Antibodypedia, 1DegreeBio)?

Check that the appropriate reference to an antibody validation profile details the antibody specified and its use in the appropriate assay/species (i.e. species used in manuscript matches species that it has been profiled for use in/reacts with) if this is the case select **'Yes.'** If for example the antibodies have been profiled in a different species or the antibody validation profile does not provide adequate information select **'No'.** If the manuscript does not describe antibodies select **'Not Applicable'.**

6. Reagents (in vitro)

6.1 is the same as for in vivo items 6.1 – see above comments.

6.2.1 Was the source of cell lines provided?

Select **'Yes'** if the manuscript describes from which source the cell lines were provided (e.g. Neonatal foreskin keratinocytes (NFSKs) were obtained directly from ATCC (PCS-200-010)). Select **'No'** if cell lines have been used in the in vitro experiment however, the source has not been provided.

If the manuscript does not describe cell lines select **'Not Applicable'.**

6.2.2 Do the authors report whether the lines used have been authenticated recently (e.g., by STR profiling: within 1 year of use)?

Select **'Yes'** if the manuscript provides a statement regarding cell authentication within a year from publication using Short Tandem Repeat (STR) profiling. Select **'No'** if cell lines have been used in the in vitro experiment however, there is no statement about cell authentication using STR profiling or if profiling has been carried out but over one year before use.

If the manuscript does not describe cell lines select **'Not Applicable'.** If the cell lines are generated in house using a referenced approach select **'Not Applicable'.**

6.2.3 Do the authors report whether the lines used have been tested for mycoplasma contamination recently (within 6 months of use)?

Mycoplasma contamination can affect cell metabolism, increase sensitivity to inducers of apoptosis or inhibit cell growth.
Select **'Yes'** if the manuscript describes if the cells have been tested for mycoplasma contamination recently (e.g. All cells were tested for mycoplasma on initial culture and at least 3 monthly thereafter using a Mycoprobe Mycoplasma Detection Kit (CUL001B, R&D systems) according to the manufacturer's instructions). Select **'No'** if cell lines have been used in the in vitro experiment however, there is no statement about testing for mycoplasma contamination.
If the manuscript does not describe cell lines select **'Not Applicable'.** If the cell lines are generated in house using a referenced approach select **'Not Applicable'.**

7. Animal information

7.1.1 Species

The commonly used names for animal taxa generally corresponds to species i.e. mouse, rat, Zebra fish, chicken, guinea-pig, non-human primates etc., the species should be reported in the manuscript, if this is done select **'Yes'** if not reported select **'No'.** It is unlikely this would be **'Not Applicable'** in the case that you believe this to be the case please explain in the comments section.

7.1.2 Strain

Different strains are often used in laboratory experiments. A mouse or a rat strain is a group of animals that is genetically uniform. Mouse strains can be inbred, mutated or genetically engineered, while rat strains are usually inbred, some examples include:

- Many mouse models are named after the gene that has been inactivated. For example, the p53 knockout mouse is named after the p53 gene which codes for a protein that normally suppresses the growth of tumours by arresting cell division and/or inducing apoptosis.
- C57BL/6, often referred to as "C57 black 6", "C57" or "black 6" (standard abbreviation: B6), is a common inbred strain of laboratory mouse.
- BALB/c is an albino, laboratory-bred strain of the House Mouse from which a number of common substrains are derived.
- Wistar as a generic name for inbred strains such as Wistar-Kyoto, developed from the Wistar outbred strains.

Select **'Yes'** if the strain has been stated and **'No'** if it has not. It is unlikely this would be **'Not Applicable'** in the case that you believe this to be the case please explain in the comments section.

7.1.3 Sex

It should be clear what the sex of the animals in each experimental group is and this should be reported as X females and / or X males. If exact numbers of a specified sex has been reported select **'Yes'** if this has not been reported an it is unclear what the sex is or how many are male/female then select **'No'.** It is unlikely this would be **'Not Applicable'** in the case that you believe this to be the case please explain in the comments section.

7.1.4 Age or weight

The exact age (can be presented as mean +/- inter quartile range) of the animals should be reported not as a range. If the exact age is reported select **'Yes (age).'** If exact values for age and weight are reported then select **'Both.'** If only an exact value for weight has been reported but not an exact age then select **'Yes (weight).'** If neither age nor weight have been reported or if only

ranges are reported select **'No'.** It is unlikely this would be **'Not Applicable'** in the case that you believe this to be the case please explain in the comments section.

7.2.1 Statement of compliance with ethical regulations

Definition Under the Act, a 'protected animal' is 'any living vertebrate, other than man, and any living cephalopod'.

This should usually be described in the methods and will include a statement about how the study was carried out in accordance/compliance with <*Name of*> guidelines, committee, institution etc. (select **'Yes'** if reported). This will vary in wording, some examples are shown below:

- Animal experimentation at the CNIO, Madrid, was performed **according to** protocols approved by the CNIO-ISCIII Ethics Committee for Research and Animal Welfare (CElyBA).
- All experiments involving mice were conducted **in accordance** to policies and procedures described in the Guide for the Care and Use of Laboratory Animals of the National Institutes of Health and were approved by the Animal Care and Use Committee at The Jackson laboratory.

If these types of statements are not reported select **'No'.**

Embryonic and fetal forms of mammals, birds and reptiles Embryonic and fetal forms of mammals, birds and reptiles are protected animals once they have reached the last third of their gestation or incubation period. Larval forms of fish and amphibians are protected animals once they are capable of feeding independently. Cephalopods are protected animals from the point when they hatch. This may be therefore be **'Not Applicable'** for experiments investigating animals before these cut-offs.

7.2.2 Committee(s) approving the experiments

This should usually be described in the methods and will include a statement about if the experiment was approved by <*Name of*> committee (select **'Yes'** if reported). This will vary in wording, some examples are shown below:

- Animal experimentation at the CNIO, Madrid, was performed according to protocols **approved by** the CNIO-ISCIII Ethics Committee for Research and Animal Welfare (CElyBA).
- All experiments involving mice were conducted in accordance to policies and procedures described in the Guide for the Care and Use of Laboratory Animals of the National Institutes of Health and **were approved** by the Animal Care and Use Committee at The Jackson laboratory.

If these type of statements are not reported select **'No'.**

It is unlikely this would be **'Not Applicable'** in the case that you believe this to be the case please explain in the comments section.

8. Human studies

8.1 Committee(s) approving the study protocol

Does the manuscript identify the committee(s) approving the study protocol?

This should usually be described in the methods and will include a statement about how the study was approved by <*Name of*> committee (select **'Yes'** if reported). This will vary in wording, some examples are shown below:

- The participants included in this study gave written informed consent and the **local ethics committee** of Region of Southern Denmark (S-20070079) **approved** the study.

If this type of statement is not reported select **'No'.** It is unlikely this would be **'Not Applicable'** in the case that you believe this to be the case please explain in the comments section.

8.2 Informed consent

Does the manuscript include a statement confirming that informed consent was obtained from all subjects?

Select **'Yes'** if the manuscript states that informed consent was obtained and **'No'** if it is not explicitly stated.

This will vary in wording and example of a statement is as follows:

- The study was approved by the regional ethical committee and written **informed consents** were obtained.
- The participants included in this study gave written **informed consent** and the local ethics committee of Region of Southern Denmark (S-20070079) approved the study.

It is unlikely this would be **'Not Applicable'** in the case that you believe this to be the case please explain in the comments section.

8.3 Consent for patient photos

For publication of patient photos, does the study include a statement confirming that consent to publish was obtained?

If there is any chance that he or she may be identified from a picture, from its legend or other accompanying text then informed is required, if the manuscript states that informed consent was obtained select **'Yes'** and **'No'** if the manuscript contains patient photos as described before and does not state that informed consent was obtained. If there are no patient photos included in the manuscript select **'Not Applicable'.**

In addition, if for example there are images such as:

- Images taken from pathology slides;
- X rays;
- Laparoscopic images;
- Images of internal organs; and
- Ultrasound images

These would be **'Not Applicable'** unless such an image is accompanied by text that could reveal the patient's identity through clinical or personal detail and therefore should have patient consent to publication.

8.4 Clinical trial registration number

Does the study report the clinical trial registration number (at ClinicalTrials.gov or equivalent)?

If the manuscript describes a human clinical trial then the clinical trial registration number should be stated, if it is select **'Yes'** if not stated select **'No.'**

If the manuscript does not describe a clinical trial select **'Not Applicable'.**

8.5 CONSORT checklist (phase II & III)

For phase II and III randomized controlled trials (RCT), does the supplementary material include a CONSORT checklist?

The main product of CONSORT is the CONSORT Statement, which is an evidence-based, minimum set of recommendations for reporting randomized trials. It offers a standard way for authors to prepare reports of trial findings, facilitating their complete and transparent reporting, and aiding their critical appraisal and interpretation. This will only be applicable if a human RCT is an element of the paper, if this is the case and a CONSORT checklist is indicated to be a supplementary material select **'Yes'.** If a phase II or III RCT is part of the manuscript but a CONSORT checklist is not included, select **'No'.**

If the manuscript does not describe a phase II or III RCT then select **'Not Applicable'.**

8.6 REMARK checklist (for tumor marker prognostic studies)

For tumor marker prognostic studies, does the supplementary material include a REMARK checklist?

The REMARK (Reporting Recommendations for Tumor Marker Prognostic Studies) guideline includes a checklist which aims to improve the reporting of these types of studies. If the manuscript describes study of human tumor marker prognostics and includes a REMARK checklist as supplementary material select **'Yes',** if it does not include this supplementary material select **'No'.** If the manuscript is not a tumor marker prognostic study then select **'Not Applicable.'**

9. Data deposition

An **accession code (or number)** is a number (possibly with a few characters in front) that uniquely identifies an entry in a database. For a public repository an author's website is not acceptable for providing this type of information.

9.1 Accession code (protein, DNA and RNA sequences)

For studies describing Protein, DNA and RNA sequences, is an accession code for data deposited in a public repository provided?

Papers reporting protein, DNA or RNA sequences (this does not include for example primers and plasmids) may report an accession number to Genbank/EMBL/DDBJ, PDB, SWISS-PROT or other appropriate, identified, publicly available database in general use in the field that gives free access to researchers from the date of initial publication.

- A sequence for the 18S rRNA gene of the strain ESV-19 of *C. neofalconis* was obtained and deposited in GenBank (accession number KT037081).
- Nucleotide sequence data have been deposited in the EMBL databases under the accession number: HE817756.

Select **'Yes'** if the accession code has been provided and **'No'** it has not. This will be **'Not Applicable'** if there are no 'protein, DNA or RNA sequences' requiring accession codes.

9.1.1 Accession code gives access to the data

Does that accession code give access to the data?

You will need to check that the accession code gives access to the data using your search engine or clicking on a link if provided. I.e. for the accession code in 9.1 you can find this by clicking on the hyperlink within the manuscript. Select **'Yes'** if the accession code gives you access to the data and **'No'** if it does not. This will be **'Not Applicable'** if there are no 'protein, DNA or RNA sequences' requiring accession codes. If the answer to 9.1 is **'No'** OR **'Not Applicable'** then select **'Not Applicable'** for this question.

9.2 Accession code (macromolecular structures)

For studies describing Macromolecular structures, is an accession code for data deposited in a public repository provided?

Papers reporting macromolecular structures may report an accession number to EMBL, PDB, or other appropriate, identified, publicly available database in general use in the field that gives free access to researchers from the date of initial publication.

Select **'Yes'** if the accession code has been provided and **'No'** it has not. This will be **'Not Applicable'** if there are no 'macromolecular structures' requiring accession codes.

9.2.1 Accession code gives access to the data

Does that accession code give access to the data?

You will need to check that the accession code gives access to the data using your search engine or clicking on a link if provided. I.e. for the accession code in 9.2 you may find this by clicking on the hyperlink within the manuscript. Select **'Yes'** if the accession code gives you access to the data and **'No'** if it does not. This will be **'Not Applicable'** if there are no 'other data' requiring accession codes. If the answer to 9.2 is '**No**' OR '**Not Applicable'** then select **'Not Applicable'** for this question.

9.3 Accession code (Crystallographic data)

For studies describing Crystallographic data for small molecules, is an accession code for data deposited in a public repository provided?

Papers reporting crystallographic structures (structure of molecules and crystals) may report an accession number to Genbank/EMBL/DDBJ, PDB, SWISS-PROT or other appropriate, identified, publicly available database in general use in the field that gives free access to researchers from the date of initial publication.

- The coordinates and structure factors for norovirus 3CL protease in complex with inhibitors have been deposited to the Protein Databank with the accession codes NVpro:17 (4XBB), NVPro:44-h (4XBC) and NVPro:44-o (4XBD).
- The atomic coordinates and structure factors have been deposited in the Protein Data Bank, www.pdb.org (PDB ID codes 4GWA, 4HAP, 4HAQ, and 4IPM).

Select **'Yes'** if the accession code has been provided and **'No'** it has not. This will be **'Not Applicable'** if there are no 'Crystallographic data' requiring accession codes.

9.3.1 Accession code gives access to the data

Does that accession code give access to the data?

You will need to check that the accession code gives access to the data using your search engine or clicking on a link if provided. I.e. for the accession code in 9.3 you can find this by clicking on the hyperlink within the manuscript. Select **'Yes'** if the accession code gives you access to the data and **'No'** if it does not. This will be **'Not Applicable'** if there are no 'Crystallographic data' requiring accession codes. If you have selected '**No'** for 9.3 this will also be '**No**'.

This will be **'Not Applicable'** if there are no 'other data' requiring accession codes. If the answer to 9.3 is '**No**' OR '**Not Applicable'** then select **'Not Applicable'** for this question.

9.4 Accession code (microarray data)

For studies describing microarray data, is an accession code for data deposited in a public repository provided?

The MGED group suggests that journals require submission of microarray data to the GEO (http://www.ncbi.nlm.nih.gov/geo/) or ArrayExpress (http://www.ebi.ac.uk/arrayexpress) databases, with accession numbers at or before acceptance of the paper for publication. An author's web site is not acceptable for providing this type of information.

A DNA microarray (also commonly known as DNA chip or biochip) is a collection of microscopic DNA spots attached to a solid surface. Scientists use DNA microarrays to measure the expression levels of large numbers of genes simultaneously or to genotype multiple regions of a genome.

An example may look like this:

- Microarray data have been deposited in the GEO database (vog.hin.min.ibcn@oeg) under accession number GSE42088.

Select **'Yes'** if the accession code has been provided and **'No'** it has not. This will be **'Not Applicable'** if there are no 'microarray data' requiring accession codes.

9.4.1 Accession code gives access to the data

Does that accession code give access to the data?

You will need to check that the accession code gives access to the data using your search engine or clicking on a link if provided. I.e. for the accession code in 9.4 you can find this by clicking on the hyperlink within the manuscript. Select **'Yes'** if the accession code gives you access to the data and **'No'** if it does not. This will be **'Not Applicable'** if there are no 'microarray data' requiring accession codes.

This will be **'Not Applicable'** if there are no 'other data' requiring accession codes. If the answer to 9.4 is **'No'** OR '**Not Applicable'** then select **'Not Applicable'** for this question.

9.5 Accession code (other data)

For studies describing other data, is an accession code for data deposited in a public repository provided?

This may include short stretches of novel sequence information such as ***epitopes*** (also known as antigenic determinant, is the part of an antigen that is recognized by the immune system, specifically by antibodies, B cells, or T cells. For example, the epitope is the specific piece of the antigen that an antibody binds to), ***functional domains*** (A protein domain is a conserved part of a given protein sequence and (tertiary) structure that can evolve, function, and exist independently of the rest of the protein chain), ***probes*** (a defined segment of DNA or RNA, usually carrying a radioactive label, used to identify a specific segment of DNA that carries the complementary base sequence), ***genetic markers*** (gene or DNA sequence with a known location on a chromosome that can be used to identify individuals or species) or ***haplotypes*** (DNA variations, or polymorphisms, that tend to be inherited together. A haplotype can refer to a combination of alleles or to a set of single nucleotide polymorphisms (SNPs) found on the same chromosome).

An example may look like this:

- A complete table for the 848 probe sets corresponding to our generated heat map including raw expression values and fold changes is available in the GEO database under accession number GSE42088.

Select **'Yes'** if the accession code has been provided and **'No'** it has not. This will be **'Not Applicable'** if there are no 'other data' requiring accession codes.

9.5.1 Accession code gives access to the data

Does that accession code give access to the data?

You will need to check that the accession code gives access to the data using your search engine or clicking on a link if provided. I.e. for the accession code in 9.5 you can find this by clicking on the hyperlink within the manuscript. Select **'Yes'** if the accession code gives you access to the data and **'No'** if it does not. This will be **'Not Applicable'** if there are no 'other data' requiring accession codes.

This will be **'Not Applicable'** if there are no 'other data' requiring accession codes. If the answer to 9.5 is **'No'** OR '**Not Applicable'** then select **'Not Applicable'** for this question.

10. Computer code

If computer code was used to generate results that are central to the paper's conclusions, authors should include a statement in the Methods section under for example ***"Code availability"*** to indicate whether and how the source code can be accessed and any restrictions.

10.1 Computer source code

Is the computer source code provided with the paper?

If the source code is explicitly provided i.e. as supplementary material select **'Yes'** if it is only referred to without being provided then select **'No'.**

10.2 Computer source code deposited in a public repository

Is the computer source code deposited in a public repository?

If the publication refers to / cites / links to an open source/public repository where the source code has been deposited select **'Yes'.** If this is not the case select **'No'.**

10.2.1 Source code accessible

Is that source code accessible?

Check if the described public repository gives access to the author's source code. If it does select **'Yes'** if however the source code cannot be easily accessed select **'No'.**

10.2.2 Function

Does the source code function as described?

Depending on your expertise only answer this question if you understand how to test the source code's functionality. If can test this select **'Yes'** or **'No'** depending on whether the source code functions as described however, if you do not know then select **'Not able to judge'.** It is unlikely this would be **'Not Applicable'** in the case that you believe this to be the case please explain in the comments section.

10.3 How can the computer source code be obtained

If NO to 10.1 and 10.2, does the manuscript indicate how the computer source code can be obtained?

For example do they state that they can provide the code if they you contact them directly. For a statement along these lines select **'Yes'**, if no statement regarding access to the source code other than refereeing to a public repository then select **'No'.** If **'Yes'** has been selected for 10.1 and 10.2 then select **'Not Applicable'.**
